# A Radical Approach To Diagnosing Infection

**DOI:** 10.1186/2197-425X-3-S1-A4

**Published:** 2015-10-01

**Authors:** N MacCallum, D Brealey, N Libert, J Pugin, M Picard-Maureau, R Sampath, D Ecker, M Singer, JL Vincent

**Affiliations:** University College London Hospitals, London, United Kingdom; Military Hospital du Val-de-Grace, Paris, France; Hopitaux Universitaires de Geneve, Geneva, Switzerland; Ibis Biosciences Abbott, Carlsbad, United States; University College London, London, United Kingdom; Erasme University Hospital, Bruxelles, Belgium

## Introduction

The cornerstone of sepsis management requires identifying the causative pathogen and initiating appropriate antimicrobial therapy. Current pathogen detection relies on culture techniques, a technology that is over 100 years old, slow and unreliably. It is not unusual for only 10% of critical care blood cultures to be positive. Due to the low yield and time taken to obtain a result, they rarely alter patient management. a novel molecular pathogen detection system, known as IRIDICA, employs polymerase chain reaction and electro spray ionisation mass spectroscopy (PCR/ESI-MS) to identify over 1000 pathogens, direct from sample without culture and within 8 hours. The RADICAL study was created to assess this technology in a real world critical care environment.

## Objectives

To compare the PCR/ESI-MS rapid pathogen detection system to hospital standard of care microbiology techniques.

## Methods

RADICAL was a multi-centre, prospective, cohort observational trial involving 9 European Critical Care Units. Critically ill patients having standard of care microbiology samples, for the investigation of potential sepsis (such as blood culture or endotracheal aspirate etc.), had a simultaneous sample for PCR/ESI-MS analysis. The sample was frozen and later analysed and results were compared to those obtained from the hospital laboratory. Although treating clinicians were blinded to the results, an independent panel of doctors reviewed the results as to whether they would have altered antibiotic prescribing.

## Results

543 patients were recruited between 2013-2014. 616 paired blood samples, 179 deep respiratory tract samples and 110 samples from other sterile sites (e.g. CSF) were obtained. a pathogen was detected by blood culture in 67 (11%) samples and in 223 (33%) samples by PCR/ESI-MS. The pathogens isolated by both techniques were those expected in the critical care environment, E.coli and S.aureas being the most frequent. The performance characteristics (Table 1) demonstrated the PCR/ESI-MS result at 8 hours had a negative predictive value of 97%.

**Table 1:** PCR/ESI-MS performance vs blood culture.

		***CULTURE***		
		POSITIVE	NEGATIVE	TOTAL
***PCR/ESI-MS***	POSITIVE	54	169	223 (36%)
	NEGATIVE	13	380	393 (64%)
	TOTAL	67 (11%)	549 (89%)	616

169 patients also had replicate blood sampling; PCR/ESI-MS was concordant in 85% of cases, culture in 55%. Relative yields from the PCR/ESI-MS were smaller for respiratory and other samples but still superior to culture and obtained within 8 hours.

The independent panel reviewed 442 case forms and concluded that the PCR/ESI-MS result could have altered antibiotic prescribing in 42% of cases, rising to 57% if the result was positive.

## Conclusions

PCR/ESI-MS performs well with numerous sample types. It is 3 times more likely to identify a pathogen in blood compared to standard culture but also carries a high negative predictive value. The PCR/ESI-MS is capable of obtaining these results within 8 hours compared to an average of 48 hours for culture. This information may be invaluable in rapidly guiding antibiotic prescribing and aiding stewardship.

## Grant Acknowledgment

RADICAL study funded by Abbott.

